# Metabolic Syndrome and Antipsychotics: The Role of Mitochondrial Fission/Fusion Imbalance

**DOI:** 10.3389/fendo.2018.00144

**Published:** 2018-04-23

**Authors:** Andrea del Campo, Catalina Bustos, Carolina Mascayano, Claudio Acuña-Castillo, Rodrigo Troncoso, Leonel E. Rojo

**Affiliations:** ^1^Departamento de Biología, Facultad de Química y Biología, Universidad de Santiago de Chile, Santiago, Chile; ^2^Escuela de Química y Farmacia, Facultad de Ingeniería, Ciencia y Tecnología, Universidad Bernardo O’Higgins, Santiago, Chile; ^3^Programa de Biología Celular y Molecular, Facultad de Medicina, Universidad de Chile, Santiago, Chile; ^4^Departamento de Ciencias del Ambiente, Facultad de Química y Biología, Universidad de Santiago de Chile, Santiago, Chile; ^5^Centro de Biotecnología Acuícola, Universidad de Santiago de Chile, Santiago, Chile; ^6^Laboratorio de Investigación en Nutrición y Actividad Física, Instituto de Nutrición y Tecnología de los Alimentos (INTA), Universidad de Chile, Santiago, Chile

**Keywords:** second-generation antipsychotic agents, mitochondrial dynamics, insulin resistance, L6 muscle cells, obesity

## Abstract

Second-generation antipsychotics (SGAs) are known to increase cardiovascular risk through several physiological mechanisms, including insulin resistance, hepatic steatosis, hyperphagia, and accelerated weight gain. There are limited prophylactic interventions to prevent these side effects of SGAs, in part because the molecular mechanisms underlying SGAs toxicity are not yet completely elucidated. In this perspective article, we introduce an innovative approach to study the metabolic side effects of antipsychotics through the alterations of the mitochondrial dynamics, which leads to an imbalance in mitochondrial fusion/fission ratio and to an inefficient mitochondrial phenotype of muscle cells. We believe that this approach may offer a valuable path to explain SGAs-induced alterations in metabolic homeostasis.

## Introduction

Second-generation antipsychotics (SGAs) are effective drugs in controlling symptoms of schizophrenia and other psychotic disorders. However, SGAs are also known to induce insulin resistance, hepatic steatosis, and accelerated weight gain, which can lead to morbid obesity in as short as 6 weeks (1). Herein, we introduce an innovative approach to explain metabolic side effects of SGAs through the impairment of the mitochondrial network morphology and insulin signaling, which results in a fusion/fission imbalance of the mitochondrial network. Our hypothesis is that the SGAs-induced disruption of the mitochondrial dynamics, manifested by a highly “fissioned” and inefficient mitochondrial network, results in a reduced capacity to trigger insulin-dependent pathways necessary to preserve adequate energy production and metabolic homeostasis. We believe that this approach may offer a valuable path to further understand the SGAs-induced insulin resistance in different tissues.

This perspective paper briefly reviews the molecular, physiological, and clinical aspects of SGAs-induced metabolic toxicity. Then, we present our explanation and initial findings on an “over-fissioned” phenotype of the mitochondrial network and impairment of the insulin receptor signaling induced by olanzapine, one of the most common drugs of the SGAs pharmacological family. We also propose, based on *in silico* simulations potential sites for the interactions between olanzapine and the extracellular domain of the insulin receptor. The purpose of this article is not only to provide definite answers on the molecular mechanisms underlying the SGAs-induced metabolic syndrome but also to present a new angle for the study of this problem, which is one of the most clinically relevant, and serious, side effect of SGAs. This perspective article offers a new insight for the development of prophylactic interventions against SGAs-induced metabolic syndrome through the screening of small molecules capable of rescuing SGAs-induced mitochondrial disruption.

## Metabolic Syndrome and Antipsychotics

The number of psychiatric patients suffering from SGAs-induced metabolic side effects continues to rise (2, 3), despite all the international guidelines for the clinical use of SGAs, which strongly suggest that this pharmacotherapy should be initiated only after a careful evaluation of basal metabolic parameters to select the appropriate drug (4). Intriguingly, in spite of their metabolic toxicity profile, clozapine, risperidone, olanzapine, quetiapine, and aripiprazole have remain among the world-top selling pharmaceuticals over the past 10 years (5, 6). Preclinical and clinical studies have shown that, among the SGAs, olanzapine is the drug with the strongest metabolic toxicity, due to its effects on weight gain (7–9), plasma glucose levels, and other metabolic parameters (10, 11).

The published evidence regarding the molecular mechanisms underlying the SGAs toxicity is still limited. However, it is known that the metabolic alterations induced by SGAs are partially mediated by hyperphagia linked to alterations in the D1/D2, 5-HT1B, 5-HT2, and 5-HT3 signaling (12), and GABA2 receptor polymorphism (13). On this regard, recent research have demonstrated the participation of serotonin signaling in glucose homeostasis through serotonylation of rab4 proteins (14), moreover other studies have shown that 5HT2 selective antagonism impairs insulin sensitivity. SGAs also induce anomalous cellular differentiation of adipocytes (15), increase lipid accumulation in the liver tissue (16), upregulate the sterol regulatory element-binding protein (17), and inhibit of the glycogen accumulation in skeletal muscle cells (18). In spite of all the current proposed mechanisms, the generation of the secondary effects of SGA is still a matter of controversy. It is important to mention that the literature describes differences of the metabolic problems presented in SGA-induced when compared with type 2 diabetes (3, 19, 20). On this regard, there is also evidence suggesting that metabolic changes due to olanzapine are tissue specific (20–23).

*In vivo* studies in rodents using the hyperinsulinemic/euglycemic clamp technique have shown that olanzapine impairs insulin sensitivity in the liver (24), skeletal muscle (21), and adipose tissue (21–23). Furthermore, a recent study showed that olanzapine decreases insulin-mediated glucose uptake through a mechanism involving an impaired hypothalamic insulin sensing during pancreatic euglycemic clamps (23). Altogether, these data seemingly confirms the results from the *in vitro* studies (16, 18) suggesting that olanzapine would induce whole-body insulin resistance. In the context of our hypothesis, it is worth mentioning that olanzapine was shown to impair lipid metabolism by increasing uptake of free fatty acids into peripheral tissues, increasing lipid oxidation in muscle cells, rising levels of long-chain 3-hydroxylated acyl-carnitines, and suppressing the respiratory exchange ratio (20). These events are indicative of an olanzapine-mediated reduced availability of fatty Acyl CoA inside the mitochondrial matrix, which would limit the supply of precursors for the tricarboxylic acids (TCA) cycle. Altogether, these results support the hypothesis that mitochondrial dysfunction plays a major effect of olanzapine-induced metabolic syndrome and the maintenance of mitochondrial homeostasis should be considered as a potential therapeutic target to prevent SGAs-induced metabolic side effects.

In spite of the relevance of skeletal muscle for the insulin-mediated conversion of glucose into ATP (25), the current literature still lacks enough mechanistic studies on the effect of SGAs on energy production and carbohydrates metabolism inside the skeletal muscle (Table 1). In order to explain our perspective, it is important to remember that the intracellular ATP is mainly produced inside the mitochondria, a highly specialized organelle involved in energy production, and that the mitochondrial energy production in skeletal muscle involves lipid metabolism, oxidative phosphorylation, and the cycle of the TCA (26). As it has been largely studied, mitochondrial function is, therefore, a sensitive indicator of the global cellular function.

**Table 1:** Mechanisms of SGA-induced metabolic side effects.

Reference	Experimental model	Molecular mechanism	Main effects
(18)	L6 rat skeletal muscle cell line	↓ Insulin-stimulated IRS-1-associated PI3K activity↓ Phosphorylation of AKT and GSK-3	↑ Glycogen synthesis
(41)	3T3-L1 cells	↓ The maximal insulin-stimulated glucose transport and lipolysis rate	Insulin resistance and alterated lipogenesis and lipolysis
(42)	Male Sprague-Dawley rats (Adipocytes INWAT and SCWAT)	↓ HSL and ↑ FAS expression	↓ Lipolytic activity
(43)	*In vitro* ligand binding assays	Affinities for anorexigenic (bombesin receptor subtype 3, calcitonin gene-related peptide receptor, cholecystokinin receptor, melanocortin-4 receptor, neurotensin receptor 1) or orexigenic (cannabinoid receptor 1, galanin 1 receptor) and high affinity for 5-HT, 5-HT2A, 5-HT2C and 5-HT6, muscarinic M1, and H1Rs	Weight gain
(44)	Human pre-adipocytes and rat muscle-derived stem cells	Activation kinase C-β (PKC-β)	Weight gain for influence adipogenic events
(45–47)	Female Sprague-Dawley rats (Arc and DVC)34 male patientsFemale Sprague-Dawley rats (coronal hypothalamic sections)	↓ Levels of POMC and ↑ NPY	Weight gain is associated with reduced appetite-inhibiting
(48)	Female Sprague-Dawley rats (Hypothalamus)	↑ Phosphorylation levels of AMPK	Weight gain and hyperphagia
(49, 50)	Female Sprague-Dawley rats (coronal sections brains)	Blockade acetylcholine (ACh) muscarinic M3 receptor (M3R)	Inhibit the acetylcholine pathway for insulin secretion
(51)	Young male patients	↑ Leptin and NPY levels	Weight gain
(52)	Male Sprague-Dawley rats (liver tissue)	↓ IRS2 levels, ↓ phosphorylation of GSK3α, and ↑ phosphorylation of GSK3β	Disturbances of glucose homeostasis (suggest an increased activity of glycogen synthase, and therefore, an increased insulin sensitivity)
(53)	Male 6-week-old ICR mice (hypothalamus)	Activates hypothalamic AMPK by antagonizing H1Rs, dopamine D2 receptors and α1-adrenoceptors	Hyperglycemia
(54)	Female Sprague-Dawley rats (liver or perirenal WAT)	↑ mRNA expression of SREBP-2 and target genes for cholesterol synthesis and transports. ↑ mRNA expression of SREBP-1c and its targeted fatty acid-related genes	Dyslipidemia
(55)	The glucose transporter from *Staphylococcus epidermidis* (GlcPSe)	The glucose transporter from *Staphylococcus epidermidis* (GlcPSe)	↓ Glucose transport
(56, 57)	Female Sprague-Dawley rats (brain; hypothalamus)	↑ Expression of HDC mRNA and ↑ the hypothalamic H1R binding; activates AMPK by blocking the H1Rs	Hyperphagia and weight gain
(58)	Female Sprague-Dawley rats (liver)	↓ AKT/GSK phosphorylation and upregulate muscarinic M3 receptors. ↑ The protein levels of SREBPs	Disturbances negative in glucose-lipid metabolic independent of weight gain
(59)	Primary human peripheral blood mononuclear cells	↓ Glucose uptake accompanied by downregulation AMPK. ↑ GLUT1 protein expression, ↓ GLUT1 mRNA expression, and GLUT1 promoter was hypermethylated. ↓ PDH complex activity	↓ Glucose uptake and affect energy metabolism
(60)	Female C57BL/6—Htr2c-null mice	Interaction with HTR2C in C57bL/6 and no interaction in Htr2c-null mice	Hyperphagia and weight gain

*IRS, insulin receptor substrate (60); PI3K, phosphatidylinositol-4,5-bisphosphate 3-kinase; AMPK, 5′ adenosine monophosphate-activated protein kinase; AKT, protein kinase B; GSK-3, glycogen synthase kinase 3; HSL, hormone-sensitive lipase; FAS, fatty acid synthase; 5-HT, serotonin or 5-hydroxytryptamine; POMC, pro-opiomelanocortin; NPY, neuropeptide Y; SREBPs, sterol regulatory element-binding proteins; HDC, histidine decarboxylase; H1R, histamine H1 receptor; PDH, pyruvate dehydrogenase; HTR2C, encodes the 5-HT 2C receptor; SGA, Second generation antipsychotic*.

## Mitochondrial Network and Metabolic Homeostasis

During the past two decades, several studies have described the functional relationship between the mitochondrial function and mitochondrial dynamics, the latter is defined as the different processes that occur to the mitochondrial network, such as fusion, fission, mitochondrial movements through the cytoskeleton, mitochondrial biogenesis, and mitophagy (27). On this regard, the proper balance between all these processes has been directly linked to a correct mitochondrial function, thus opening new possibilities for regulating the mitochondrial metabolism through the pharmacological interventions of mitochondrial dynamics.

Impaired insulin signaling and mitochondrial dysfunction are two clear signs of abnormal metabolic response in skeletal muscle cells. Given these characteristics, several studies have reported morphological differences of the mitochondrial network in obese and diabetic patients (28), but none of these studies have looked at mitochondrial dynamics in SGAs users. Interestingly, an *in vitro* study, by Contreras-Shannon et al., showed that Clozapine, a member of the SGA family, alters mitochondrial morphology and ATP levels in cultured insulin-responsive cells in a dose-dependent manner (29).

The relationships between mitochondrial dynamics and insulin physiological responses in skeletal muscle cells are an active field of research, to which we have contributed by describing how insulin promotes mitochondrial fusion after in cardiac myocytes and L6 muscle cells (30). We also reported that the regulation of mitochondrial morphology toward incomplete fusion impairs insulin signaling and glucose uptake in L6 myoblasts (31).

Previous results suggested a direct influence of SGAs on the peripheral insulin resistance. Particularly Engl et al. demonstrated that olanzapine impairs glycogen synthesis by disrupting insulin signaling in a model of L6 skeletal muscle cells (18). Ardizzone and coworkers observed that SGAs inhibit glucose transport in L6 myoblasts (32). These latter *in vitro* studies suggest that SGAs would induce insulin resistance, although the concentrations of olanzapine used for these studies exceed those observed in human plasma (33). However, these preliminary *in vitro* studies suggesting insulin resistance induced by SGAs were confirmed and further characterized in rats by Martins and coworkers (34), who demonstrated that olanzapine administered directly to the CNS induces the expression of hypothalamic 5′ adenosine monophosphate-activated protein kinase and hepatic insulin resistance, suggesting a CNS target for the metabolic dysregulation of atypical antipsychotics. Another confirmation came from a clinical study demonstrating that only 9 days of oral olanzapine treatment causes significant elevations in postprandial insulin, glucagon-like peptide 1, and glucagon coincident with insulin resistance (35). According to Teff et al. aripiprazole, another SGAs drug, would also induce insulin resistance.

In view of these findings, we decided to study effects of SGAs through the alterations of the mitochondrial dynamics induced by olanzapine and also to perform an *in silico* search for potential interactions between the insulin receptor and olanzapine. We believe this model system could help to further explain the multicellular metabolic toxicity of SGAs.

We also examined the question as to whether olanzapine would interact with residues of the extracellular domain of the insulin receptor. It is worth mentioning that the insulin receptor signaling pathways are involved in the peripheral mechanisms of SGAs-induced toxicity (3). We modeled the human insulin receptor (36) using MODELLER 9.14 (37) the crystal structure (PDB ID: 3W14) (38) with an identity of 99%. The best model obtained was refined using Charmm 33b1 with the conjugate gradient.

Dockings of olanzapine (http://zinc.docking.org/; olanzapine code: 52957434) and model of human insulin receptor were performed with the AutoDock4 package (39), using a Lamarckian algorithm and assuming total flexibility of all compounds studied. The grid maps were made up of 40 × 40 × 40 points, with a grid-point spacing of 0.375 Å of the center of the molecule. The AutoTors option was used to define the ligand torsions, and the docking results were then analyzed by a ranked cluster analysis, resulting in conformations with the highest overall binding energy (largest negative −ΔGB value).

**Figure 1:**
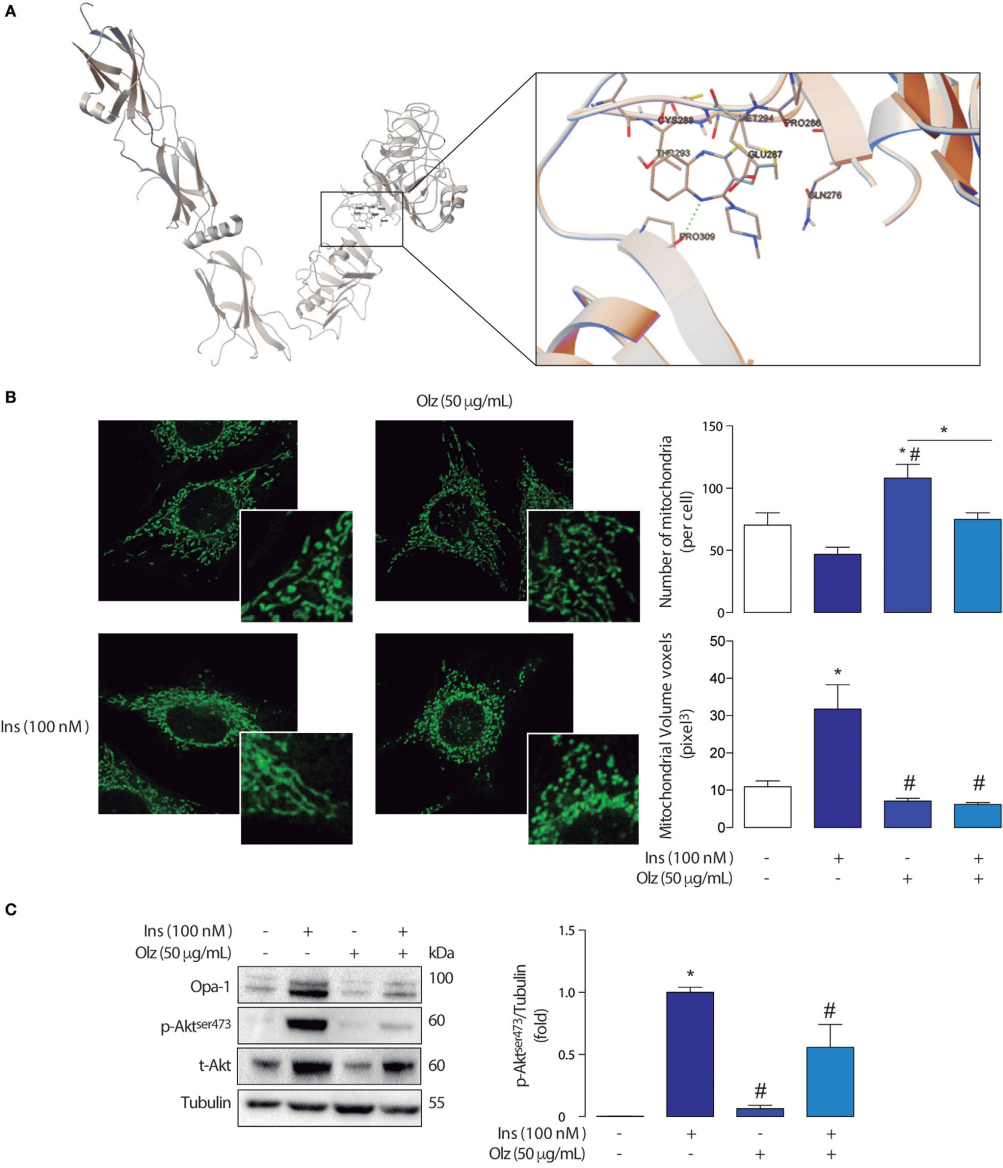
**(A)** Graphical representation of the interactions sites for olanzapine and the extracellular domain of de insulin receptor according to Docking Studies. The model of human insulin receptor and the structure of olanzapine were obtained using the AutoDock4 package. **(B)** Mitchondrial morphology of L6 myotubes and densitometry of confocal micrographies. Mitochondrial morphology in control conditions (left) and the olanzapine 160 µM treated cells (right) showing fragmentation of the mitochondrial network. *p < 0.05 vs control ^#^*p* < 0.05 vs Ins. **(C)** Detection of insulin signaling proteins from L6 myotubes treated with olanzapine and insulin. The mitochondrial immunoblots for Opa-1, Akt, and p-Akt in L6 myotubes treated with olanzapine (OLZ) and Insulin (Ins). *p < 0.05 vs control ^#^*p* < 0.05 vs Ins.

We observed that olanzapine displayed a binding energy of −6.89 kcal/mol located at H bond near to Pro309 (2.89 Å). Other interacting residues around the olanzapine were Gln276, Glu287, Cys288, Thr293, and Met294 (Figure 1A). Both, binding energy and interatomic distances suggest there are potential interactions between olanzapine and the extracellular domain of the insulin receptor. This is of course susceptible of experimental verification through binding experiments with radiolabeled ligands, directed mutagenesis of the insulin receptor, or through surface plasmon resonances studies.

The changes in mitochondrial morphology were assessed in L6 myoblasts incubated with 400 nM MitoTracker green in Krebs solution for 25 min and then washed with Krebs solution for 5 min. Confocal images stacks of the mitochondrial network were captured with a Nikon C2 Confocal microscope, using a 60× Plan-Apochromatic λ CFI oil (1,4) objective, as described by del Campo et al. (31). Z-stacks were deconvolved, thresholded, and 3D-reconstructed using ImageJ software (NIH). Number and volume of individual mitochondria were quantified using the 3D Object Counter plug-in.

The analyses of mitochondrial network assessed through variations in mitochondrial number and volume in myoblasts treated with olanzapine showed that this drug induces mitochondrial fragmentation when compared with control (no treatment) myoblasts (Figure 1B). This fragmented phenotype is determined by a significant increase in the number of mitochondria per cell accompanied by a decrease in the volume of each mitochondrion (voxels = pixels^3^).

Interestingly, olanzapine also disrupted the effects of insulin in mitochondrial dynamics (Figure 1B). As shown in previous studies, insulin 100 nM promotes mitochondrial fusion in L6 myoblasts, our results support the fact that a fused-like phenotype, given by a significant increase in the volume of mitochondria and a reduced number of mitochondria per cell, can be found in myoblasts treated with insulin. On the basis of these previous findings, we investigated whether the pre-incubation of olanzapine impairs the mitochondrial fusion promoted by insulin 100 nM. Our results showed that the pre-incubation with olanzapine actually impairs the action of insulin on the mitochondrial dynamics by significantly decreasing the mitochondrial volume, compared with cells incubated only with insulin 100 nM 3 h only (Figure 1B). Each experiment was repeated at least three times. One-way ANOVA was used as statistic test and a subsequent Tuckey post-test was applied, statistical significance was defined as *p < 0.05.

These results show that olanzapine affects the mitochondrial network probably promoting mitochondrial dysfunction on its own, not only by inducing mitochondrial fragmentation but also by interrupting insulin-mediated changes of the mitochondrial network in skeletal muscle cells. The mitochondrial fragmentation on its own impairs insulin signaling, as proven by the use of antisense adenovirus toward Mfn2 and microRNA toward OPA-1 in L6 skeletal muscle cells, decreases in Akt phosphorylation (31). We observed that olanzapine not only promotes a fragmented phenotype of the mitochondrial network but also inhibits insulin-mediated fusion and decreases Akt phosphorylation (Figure 1C). This is a clear indicative that metabolic alterations induced by olanzapine are related with the fragmentation of the mitochondrial network and mitochondrial metabolic dysfunction. This data is an indicative that olanzapine would limit the utilization of different molecules to produce cellular ATP, which subsequently could lead to maladaptation of the skeletal muscle.

## Alterations in the Expression of Mitochondrial Fusion Proteins and Insulin Signaling

Opa-1 is a mitochondrial protein involved in inner mitochondrial membrane fusion and maintenance of mitochondrial cristae (40). As previously reported a 3 h insulin stimulus promotes an increase of Opa-1 protein levels through the Akt-mTOR-NFkB-Opa-1 signaling pathway promoting mitochondrial fusion (30). A mitochondrial fusion-like phenotype has been associated with healthy metabolic homeostasis in eukaryotic cells (27, 30, 31). Interestingly, olanzapine decreases the insulin-induced expression of Opa-1 in L6 cells treated with insulin for 3 h. More specifically, we observed that in L6 skeletal muscle cells insulin (100 nM 3 h) induced an increase of long and short isoforms of Opa-1, which was impaired by olanzapine (Figure 1C). These results suggest that olanzapine disrupts insulin signaling, through a decrease in Akt phosphorylation, and also impairs the subsequent modification of mitochondrial dynamics, contributing to mitochondrial dysfunction.

## Concluding Remarks

Based on our data and previous evidence in this field (29, 40), we here propose a new perspective to explore the mechanism of SGAs metabolic toxicity based on the impairment of the mitochondrial dynamics, which could explain the development of accelerated metabolic syndrome manifested by insulin resistance, weight gain, lipid accumulation, and hyperglycemia. In other words, the metabolic disturbances induced by SGAs affect one of the most fundamental functions of living cells, which is ATP production in the mitochondria.

## Author Contributions

AC performed experiments and data analysis, CB performed skeletal muscle experiments, CM designed and performed Autodock experiments, CA-C designed experiments and contributed to the manuscript writing, RT summarized SGA studies in Table 1, and LR outlined the manuscript, overviewed the experiments, analyzed data, and wrote the manuscript.

## Conflict of Interest Statement

The authors declare that the research was conducted in the absence of any commercial or financial relationships that could be construed as a potential conflict of interest.
